# Donepezil and life expectancy in Alzheimer’s disease: A retrospective analysis in the Tajiri Project

**DOI:** 10.1186/1471-2377-14-83

**Published:** 2014-04-11

**Authors:** Kenichi Meguro, Mari Kasai, Kyoko Akanuma, Mitsue Meguro, Hiroshi Ishii, Satoshi Yamaguchi

**Affiliations:** 1 Division of Geriatric Behavioral Neurology, CYRIC, Tohoku University, Sendai, Miyagi 980-8575, Japan; 2 The Osaki-Tajiri SKIP Center, Osaki, Miyagi 989-4413, Japan

**Keywords:** Alzheimer’s disease, Donepezil, Cholinesterase inhibitors, Life expectancy, Nursing home

## Abstract

**Background:**

Cholinesterase inhibitors (ChEIs) such as donepezil have the effect of delaying progression of Alzheimer’s disease (AD), but their effect on life expectancy is unclear. We analyzed the influence of donepezil on life expectancy after onset of AD, together with the effects of antipsychotic drugs and residency in a nursing home.

**Methods:**

All outpatients at the Tajiri Clinic from 1999–2012 with available medical records and death certificates were included in a retrospective analysis. The entry criteria were a dementia diagnosis based on DSM-IV criteria and diagnosis of AD using NINCDS-ADRDA criteria; medical treatment for more than 3 months; and follow up until less than 1 year before death.

**Results:**

We identified 390 subjects with medical records and death certificates, of whom 275 had a diagnosis of dementia that met the entry criteria. Of 100 patients diagnosed with AD, 52 had taken donepezil and 48 patients had not received the drug due to treatment prior to the introduction of donepezil in 1999 in Japan. The lifetime expectancies after onset were 7.9 years in the donepezil group and 5.3 years in the non-donepezil group. There was a significant drug effect with a significant covariate effect of nursing home residency. Other covariates did not reach a significant level.

**Conclusions:**

Although this report has the limitation of all retrospective analyses: the lack of randomization, we found a positive effect of donepezil on lifetime expectancy after onset of AD. This may be due to a decreased mortality rate caused by reduction of concomitant diseases such as pneumonia. The similar life expectancies in patients taking donepezil at home and those not taking donepezil in a nursing home indicated a positive health economic effect of the drug.

## Background

Several longitudinal studies have shown that cognitive impairment with advancing age is a negative predictor of subsequent survival [1]. This association remains after adjusting for medical conditions and self-rated health, and thus has been attributed to the effects of decreased biological vitality [2]. However, it has also been suggested that the cognition-mortality link reflects more than just a reduction in biological vitality. Systematic reviews have concluded that the terminal decline is a multifactorial phenomenon, with origins that operate across the entire lifespan [3].

Higher levels of cognition are associated with better health literacy and higher socioeconomic status, and lead to better health management and reduced mortality. Education is positively associated with access to health services, increased likelihood of correctly following instructions for use of medication, and better chronic disease management [4]. Health literacy may, therefore, result in earlier diagnosis and earlier intervention, thus reducing disease impact on cognitive development over the lifespan. Alternatively, possible positive effects of psychosocial activities such as exercise and mental activities may be decreased by cognitive impairment. In addition to these effects, dementia itself is a risk factor for decreased life expectancy.

Life expectancy for patients with dementia directly influences prevalence and service needs and is a common question posed by families and patients. A recent [5] systematic review compared mortality and survival in dementia with estimated life expectancies in the general population. Survival after diagnosis of dementia varies considerably and depends on numerous factors and complex interactions among these factors. Relative loss of life expectancy decreases with age at diagnosis and also depends on gender, dementia subtype, and severity stage. A definitive meta-analysis of survival in dementia is precluded by deficiencies in primary studies.

Alzheimer’s disease (AD) is the main cause of dementia. At present, there are no curative drugs for AD; however, symptomatic drugs such as cholinesterase inhibitors (ChEIs) or memantine may delay progression of the disease. This effect combined with psychosocial interventions may increase quality of life (QOL) [6]. Beyond delayed progression and increased QOL, the ultimate outcome of drug treatment should be measured in terms of life expectancy.

ChEIs such as donepezil are used for symptomatic treatment of AD. Treatment with these drugs can delay nursing home placement [7], reduce the caregiver burden and the time spent caring [8], and possibly reduce mortality for patients living in nursing homes [9] and in the community [10]. However, the effect on mortality is uncertain: Lopez et al. [10] found that ChEIs can delay a move to a nursing home, but have no effect on life expectancy, whereas a recent cohort study [11] in 7,073 AD patients in the Swedish Dementia Registry suggested that ChEIs were associated with a lower risk of death and myocardial infarction. These associations were stronger with increasing ChEI dose, which may be due to the vagotonic and antiinflammatory effects of these drugs on atherosclerosis.

In this study, we examined whether donepezil has an effect on life expectancy in AD. We hypothesized that 1) the drug has a positive effect on life expectancy in AD, 2) that nursing home residency also has a positive effect, and 3) that use of antipsychotic drugs has a negative effect. We analyzed donepezil alone because in Japan this drug has been used since 1999, whereas other drugs such as galantamine or memantine have only been used since 2011. The combined effect of donepezil and nursing home residency was also analyzed. Although the retrospective design, this is the long-term study of the possible effect of donepezil on life expectancy of patients with AD.

## Methods

### Patients

All patients were outpatients at the memory clinic at the Osaki-Tajiri SKIP Center or residents at the Kagobo-no-sato nursing home, which is associated with the Osaki-Tajiri SKIP Center. All nursing home patients received medical services at the memory clinic. Outpatients and nursing home patients all underwent magnetic resonance imaging (MRI) to confirm the medical diagnosis of dementia. Chest X-ray, electrocardiogram, and blood tests were performed to exclude possible systemic diseases that could affect cognitive functions. The entry criteria were 1) a dementia diagnosis based on DSM-IV criteria, a Clinical Dementia Rating (CDR) [12] of 1+, and diagnosis of dementing diseases using the established criteria described below; 2) medical treatment for more than 3 months; and 3) follow up until less than 1 year before death.

### Dementia diagnosis

Diagnoses of the following diseases were made at a meeting of two neurologists, a psychiatrist, and a physician.

1) Pure AD without cerebrovascular diseases (CVD) was diagnosed in patients who met NINCDS-ADRDA criteria for probable AD [13] and had no CVD on MRI. On MRI, low signal intensity on T_1_-weighted images, high signal intensity on T_2_-weighted images, and high signal intensity surrounding the low signal intensity areas on FLAIR images were considered to show CVD.

2) AD with CVD was diagnosed based on NINCDS-ADRDA criteria for probable AD and on evidence for the presence of CVD on MRI; however, CVD lesions were judged to be concomitant with AD and not responsible for cognitive deterioration.

3) VaD was diagnosed based on NINDS-AIREN criteria for probable VaD [14].

4) Dementia with Lewy bodies (DLB)/ Parkinson disease with dementia (PDD) and frontotemporal lobar degeneration (FTLD) were diagnosed based on the respective consensus criteria [15,16].

5) Others conditions were diagnosed in 20 patients with head trauma (n = 3), hydrocephalus (n = 3), diabetic dementia (n = 2), vitamin B_12_ deficiency (n = 2), thyroid dysfunction (n = 2), progressive supranuclear palsy (n = 1), alcoholic dementia (n = 1), chronic subdural hematoma (n = 1), hepatic encephalopathy (n = 1), renal encephalopathy (n = 1), hypoxic encephalopathy (n = 1), syphilis (n = 1), and brain tumor (n = 1).

### Analyses

The main outcome was the time (months) between onset of AD and death. Age at the first clinic visit, gender, presence of concomitant CVDs, use of antipsychotic drugs (typical and atypical), and nursing home residency were analyzed as covariates.

One-way Analysis of Variance (ANOVA) was performed to analyze the drug effect with the covariate effects. A Spearman correlation was calculated between the period of donepezil administration and life expectancy.

Written informed consent was obtained from each of the patients and from the family of those with dementia at entry according to the Declaration of Helsinki (BMJ 1991; 302: 1194). The study was approved by the ethical committee of Tohoku University Graduate School of Medicine, as well as those of the Osaki-Tajiri SKIP Center.

## Results

### Dementing diseases

**Figure 1:**
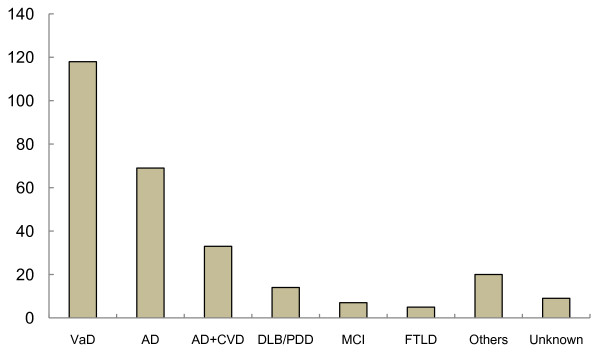
**Numbers of patients with different dementing diseases.** VaD = vascular dementia, AD = Alzheimer’s disease, CVD = cerebrovascular diseases, DLB = dementia with Lewy bodies, PDD = Parkinson disease with dementia, MCI = mild cognitive impairment, FTLD = frontotemporal lobar degeneration. The vertical line indicates the numbers.

A total of 390 medical records and death certificates were available for the period 1999–2012, including 275 patients with diagnoses of dementia that met the entry criteria. The number of patients with each dementing disease is shown in Figure 1. The most common disease was VaD, followed by AD and AD with CVD. Of the 100 patients with AD and AD with CVD (both of which are diseases that can be treated with donepezil), 52 received donepezil and 48 patients did not receive the drug due to treatment prior to the introduction of donepezil in 1999 in Japan.

### Demographics and causes of deaths

The patients who received donepezil (DNP group, n = 52) included 18 men and 34 women, and had a mean (SD) age of 80.5 (6.9) years old and a mean score on the Mini-Mental State Examination (MMSE) of 14.1 (6.3). The patients who did not receive donepezil (non-DNP group, n = 48) included 10 men and 38 women, and had a mean age of 82.9 (6.7) years old and a mean MMSE score of 14.1 (6.8). Thus, there was no significant difference in the male-to-female ratio, age or MMSE score between the two groups. The two groups also had a similar distribution of causes of death (Table 1), with pneumonia and respiratory failure being the main causes in each group.

**Table 1:** Demographics and causes of death for DNP and non-DNP groups

	**DNP group**	**Non-DNP group**
n (men/women)	52 (18/34)	48 (10/38)
Mean age (SD), years	80.5 (6.9)	82.9 (6.7)
Causes of death		
Pneumonia and/or respiratory failure	19	21
Cancer	1	4
Stroke	3	3
Heart diseases	5	5
Others	13	10
Unknown	11	5

DNP = donepezil.

There were no group differences for men/women ratio, causes of deaths (chi-square tests) and mean age (t-test).

### Effect on life expectancy

**Figure 2:**
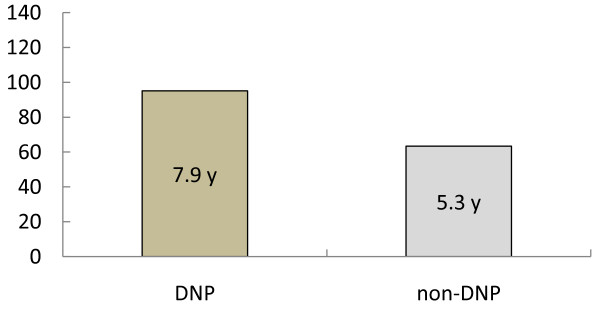
**Donepezil effect on life expectancy in AD.** DNP = donepezil, AD = Alzheimer’s disease. The vertical line indicates life expectancy (months). There was a significant drug effect (F = 14.497; p < 0.001) with a significant covariate effect of nursing home residency (F = 18.167. p < 0.001). No other covariates reached a significant level (age: F = 0.075, p = 0.785; gender: F = 0.171, p = 0.680; CVD: F = 3.827, p = 0.054; typical antipsychotic drugs: F = 0.353, p = 0.554; atypical antipsychotic drugs: F = 0.583, p = 0.447).

The life expectancies after onset were 7.9 years in the DNP group and 5.3 years in the non-DNP group (Figure 2). There was a significant drug effect with a significant covariate effect of nursing home residency. Other covariates did not reach a significant level.

**Figure 3:**
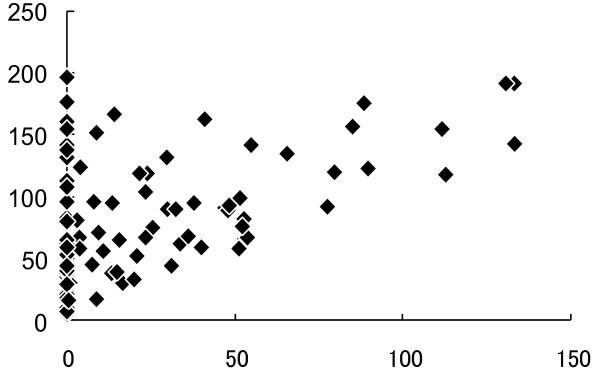
**Period of donepezil use and life expectancy in AD.** AD = Alzheimer’s disease. There was a significant Spearman correlation (Rs = 0.439, p < 0.0001) between the period of donepezil administration (months, horizontal line) and life expectancy (months, vertical line).

Spearman analysis showed a significant positive correlation between the period of donepezil use and life expectancy in AD (Figure 3).

Since there was a significant effect of nursing home residency on life expectancy, the patients were classified into 4 groups: those taking donepezil and living in a nursing home (DNP + NH group) or at home (DNP + non-NH group), and those not taking donepezil and living in a nursing home (non-DNP + NH group) or at home (non-DNP + non-NH group). The demographics of these groups are shown in Table 2. There were no group differences for men/women ratio and mean age.

**Table 2:** Demographics for four groups

	**DNP & NH group**	**DNP & non-NH group**	**Non-DNP & NH group**	**Non-DNP & non-NH group**
N	10	42	14	34
Men/Women	1/9	17/25	2/12	8/26
Age (mean)	80.2	80.6	82.9	82.9
(SD)	8.8	6.5	6.4	6.8

DNP = donepezil, NH = nursing home.

There were no group differences for men/women ratio (chi-square tests) and mean age (one-way ANOVA).

**Figure 4:**
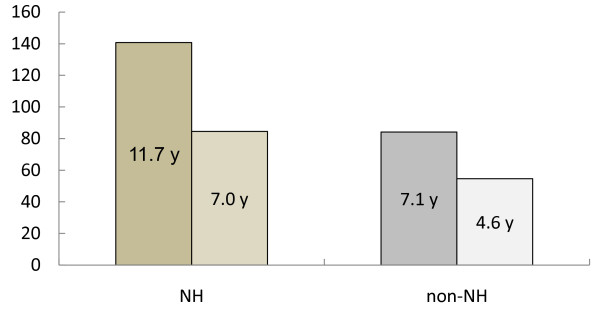
**Donepezil and nursing home effects on life expectancy in AD.** NH = nursing home, AD = AD = Alzheimer’s disease. The vertical line indicates life expectancy (months). From the left to right, DNP + NH group, non-DNP + NH group, DNP + non-NH group, and non-DNP + non-NH group. There were significant effects of the drug (F = 14.105; p < 0.001) and nursing home residency (F = 17.326, p < 0.001) without significant interactions (F = 0.894, p = 0.347). No other covariates reached a significant level (age: F = 0.206, p = 0.651; gender: F = 0.796, p = 0.375; CVD: F = 2.911, p = 0.091; typical antipsychotic drugs: F = 0.035, p = 0.852; atypical antipsychotic drugs: F = 0.362, p = 0.549).

As shown in Figure 4, life expectancy was increased by nursing home residency, in addition to the donepezil treatment effect.

## Discussion

This report demonstrates the possible effect of donepezil on life expectancy after onset of AD. This possible effect was found to be independent of age, gender, and the use of antipsychotic drugs. Before discussing the results, we should note methodological limitations.

### Limitation of the study

This report has the limitation of all retrospective analyses: the lack of randomization. The evaluation of a drug effect is essentially dependent on how patients are ascertained to the drug or to non-drug groups. However, observational studies can help understanding associated effects of drugs, since main bias is carefully considered. We considered that there were no remarkable differences between DNP group and Non-DNP group for social status, such as economic status, or family supports. Actually the patients were all residents in Tajiri, a typical agricultural town, where they were born, grew up, and got married in the same area. All men analyzed were farmers and all women analyzed were house wives, having similar life styles. This unique “pure” social status can exclude possible confounding effects of social factors on life expectancy.

Regarding health status, two groups’ distribution of vascular risk factors (hypertension, diabetes mellitus, dyslipidemia, cardiac diseases) were not statistically different (chi-square tests, data not shown). Also, we previously reported the Quality-Adjusted Life-Year (QUALY) for various degrees of daily activities of AD [17]; we herein analyzed the QUALY for the DNP and Non-DNP groups, and the same results were obtained (data not shown). Thus we think that although the study has the limitation, the results can provide useful information on the life expectancy in AD. The possible reasons for the positive finding were discussed below.

### Cause of death

Knowledge of the causes of death is of value in terminal care of patients with dementia. Cancer, heart disease, and stroke are the main causes of death in the whole Japanese population (the Ministry of Health, Labour and Welfare 2011 < http://www.mhlw.go.jp/toukei/saikin/hw/jinkou/suikei10/index.html >), but not in patients with AD. Stroke and heart diseases are vascular diseases that are commonly accompanied by VaD, but not by AD. In contrast, respiratory failure or pneumonia is common in patients with AD. In cases with clinical and pathological diagnoses of dementia and a complete autopsy, Brunnström et al. [18] found that the two most common causes of death were bronchopneumonia and ischemic heart disease, while cancer was uncommon. Pneumonia as an immediate cause of death in dementia may reflect a terminal stage in which patient care and feeding is difficult to manage effectively.

Wada et al. [19] found that use of antipsychotics, presence of CVD in the basal ganglia, severity of dementia, and male gender were associated with aspiration pneumonia in AD. However, our investigation of these factors did not show these relationships in patients who did and did not take donepezil (data not shown). Drugs such as angiotensin-converting enzyme inhibitors improve the swallowing reflex, thus preventing exacerbation of pneumonia [20], but we also found no effect of these drugs (data not shown). Thus the longer life expectancy in the DNP group was considered to be due to donepezil itself.

### Why does donepezil prolong life expectancy?

As described above, the possible effect of donepezil on mortality is uncertain. A recent cohort study [19] showed that donepezil use was associated with a lower risk of death and myocardial infarction, probably because of the vagotonic and anti-inflammatory properties of the drug on atherosclerosis. Given the important actions of ChEIs on the heart, Sato et al. [21] undertook a retrospective cohort investigation to assess the effects of donepezil on cardiovascular mortality. Contrary to the drug action and the higher risk for sinus node dysfunction or cardiac conduction impairment, this analysis showed better cardiovascular and overall survival in donepezil-treated patients. This finding is supported by an animal study showing that oral donepezil improved survival in a mouse congestive heart failure model through prevention of pumping failure and cardiac remodeling [22]. However, in our AD patents, there was no difference in heart disease between the DNP and non-DNP groups.

Donepezil may have a negative effect on aspiration pneumonia due to a side effect of nausea. An increased gastro-esophageal reflex may induce pneumonia. This and the absence of an effect on heart disease in our patients suggest that the effect of donepezil on life expectancy was not purely pharmacological. Single photon emission CT studies have shown increased psychomotor speed or attention function after administration of donepezil, associated with frontal, limbic, lower temporal lobes in the cingulate cortex [23] or frontal and parietal lobes in the basal ganglia [24]. Stimulation of psychomotor speed and attention by donepezil is consistent with the higher mortality in older adults with lower perceptual speed [1].

The effect of donepezil of delaying progression of AD may also maintain the “energy level” of life. Patients with moderate to severe AD show instrumental and basic activities of daily living (ADL) benefits after donepezil administration. In a study of the long-term effects of donepezil on the use of community-based home help service, Wattmo et al. [25] found that the drug reduced the use of the service, i.e., maintained higher self-supported levels of instrumental ADL. Psychosocial activities may occur more smoothly with maintenance of ADL, as well as with increased psychomotor speed and attention. Rehabilitation also has a long-term effect in decreasing mortality, and especially improves motor disability and ADL [26] and prevents aspiration pneumonia [27]. Based on these observations, a prospective longitudinal study is needed to clarify the effects of donepezil in patients with AD.

### Effect of antipsychotics on life expectancy

In recent years, atypical antipsychotic drugs such as risperidone have largely replaced conventional medications such as haloperidol due to equal efficacy and better tolerance [28]. In particular, physicians prefer to prescribe atypical antipsychotics to patients with dementia. However, atypical antipsychotics were found to have efficacy limitations for treatment of BPSD in a double-blind randomized placebo-controlled trial [29].

Such limitations are not the only problem with antipsychotics, since these drugs have a negative effect on life expectancy [30] and this has led to a discussion of the appropriateness of their use. A large retrospective cohort study (n = 4,369) [31] showed twofold and fivefold increases in mortality in users of atypical and conventional antipsychotics, respectively, compared to non-users. Our results were in contrast to these findings, but this was probably because the AD patients took only small doses of antipsychotic drugs (risperidone 1 mg/day, levomepromadine 5 mg/day) for less than 3 months.

### Nursing home effect

The effect of nursing home residency should also be considered. Compared with living at home, better management of therapy (better drug compliance) and a better general environment (good temperature and nutrition) in a nursing home may have a positive effect on life expectancy. However, the similar life expectancies of patients taking donepezil at home and those who did not receive donepezil and lived in a nursing home suggests a positive health economics effect of the drug. In the Long-Term Care Insurance (LTCI) system in Japan, nursing home residency costs about 100,000 Yen/month, whereas donepezil treatment costs 500 Yen/day, i.e., 15,000 Yen/month. Thus, use of donepezil at home can significantly reduce the cost of management of patients with AD.

## Conclusions

Although this report has the limitation of all retrospective analyses: the lack of randomization, we found a positive effect of donepezil on lifetime expectancy after onset of AD. This may be due to a decreased mortality rate caused by reduction of concomitant diseases such as pneumonia. The similar life expectancies in patients taking donepezil at home and those not taking donepezil in a nursing home indicated a positive health economic effect of the drug.

## Competing interests

We declare that we have no financial competing interests or non-financial competing interests.

## Authors’ contributions

KM: data analysis and writing an article. MK: data analysis. KA, MM: data collection. HI, SY: physicians in charge and data collection. All authors read and approved the final manuscript.

## Pre-publication history

The pre-publication history for this paper can be accessed here:

http://www.biomedcentral.com/1471-2377/14/83/prepub
